# Verrucous Hemangioma

**Published:** 2019-01-07

**Authors:** Katie Laun, Jake Laun, David Smith

**Affiliations:** ^a^Department of Emergency Medicine, Florida Hospital Orlando, Orlando; ^b^Department of Plastic Surgery, Morsani College of Medicine, Tampa, Fla

**Keywords:** verrucous hemangioma, verrucous, hemangioma, malformation, capillary hemangioma

**Figure:**
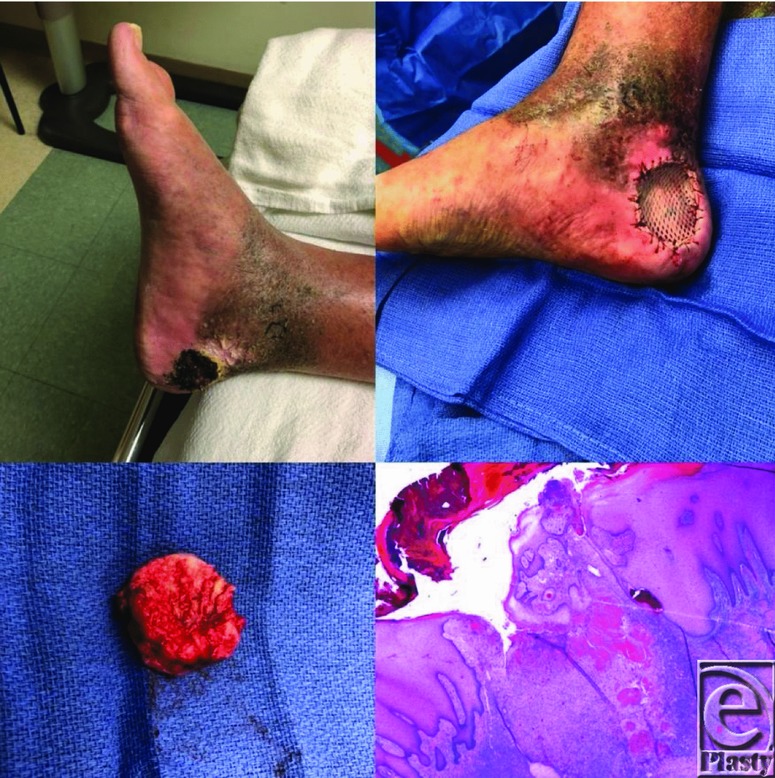


## DESCRIPTION

A 66-year-old man presented with a lesion on his right medial heel that had been present since birth. He thought the lesion was a birthmark but stated that it had recently started to change and became progressively enlarged. The lesion was biopsied and confirmed to be a verrucous hemangioma. It was completely excised, and the defect was reconstructed with a split-thickness skin graft.

## QUESTIONS

1. What is verrucous hemangioma?
2. How is a verrucous hemangioma diagnosed?
3. What are the treatment options for verrucous hemangioma?
4. What is the prognosis for verrucous hemangioma?

## DISCUSSION

Verrucous hemangiomas are uncommon capillary or cavernous hemangiomas that are associated with reactive skin changes such as hyperkeratosis and papillomatosis.^1^ They have been referred to by many names in the literature until 1967 when they were described as “congenital vascular malformation comprising a capillary or cavernous hemangioma in the dermis and subcutaneous tissue associated with reactive epidermal acanthosis, papillomatosis, and hyperkeratosis, distinguishing it from angiokeratoma.”^2^^(p1-2)^ The exact incidence is difficult to determine as it has been referred to by many different names in the past.^3^ They can be present since childhood or appear later in life during adulthood and are usually found on the lower extremities, being unilateral in approximately 95% of cases.^1^^,^^4^ There is no clear classification of these lesions, but they appear similar to vascular neoplasms (positive for glucose transporter type-1 [GLUT-1] and Wilms tumor-1 [WT1]).^5^ GLUT-1 functions to transport glucose across cells and is often aberrantly expressed in tumors. WT1 gene is a tumor suppressor gene that plays an important role in angiogenesis by affecting vascular endothelial growth factor (VEGF) and vascular smooth muscle and has shown to be involved in vascular anomalies if defective.^6^

Verrucous hemangiomas are usually diagnosed via histopathology but can have a characteristic physical appearance as well. Initially, they can appear as a nonkeratotic, blue-red lesion that progressively enlarges and becomes more hyperkeratotic and verrucous in nature, especially after infection or subsequent trauma.^1^^,^^5^ They can range in diameter from 4 mm to more than 8 cm.^4^ Histologically, they show “hyperkeratosis, variable epidermal acanthosis, and papillary telangiectasias overlying a deep cavernous or capillary hemangioma.”^1^^(p2)^ Verrucous hemangiomas must be differentiated from angiokeratomas that do not extend as deep into the reticular dermis and subcutaneous adipose tissue as verrucous hemangiomas. This deeper extension has implications in the surgical resection of the lesions, which must extend deeper than when excising angiokeratomas.^7^^,^^8^

Verrucous hemangiomas should be identified, diagnosed, and treated as early as possible to limit the extent of resection. Because of the risk of recurrence, resection should encompass the deep portions of the lesion with usually a 1-cm margin of excision. If the lesion is small (<2 cm), cryosurgery, electrocautery, or laser therapy can be used but resection is the principal treatment. These additional therapies can be used in combination with resection for extensive lesions to further assist in reducing the risk of recurrence.^1^

The prognosis for verrucous hemangioma is good, with recurrence being low when adequate surgical margins are utilized and if in combination with additional therapies as stated earlier.^1^ If inadequate wide excision is performed, recurrence can exceed 30%.^4^ Verrucous hemangiomas are benign lesions, and surveillance is mainly performed for local recurrence.

Verrucous hemangiomas are rare cutaneous lesions that appear as a verrucous lesion, principally on one of the lower extremities. They enlarge over time, and it is important to identify and resect them early to limit the amount of resection as well as decrease the risk of recurrence that can be high if a deep enough resection is not performed.
